# High-dose-rate brachytherapy for soft tissue sarcoma in children: a single institution experience

**DOI:** 10.1186/1748-717X-3-9

**Published:** 2008-04-19

**Authors:** Gustavo A Viani, Paulo E Novaes, Alexandre A Jacinto, Celia B Antonelli, Antonio Cassio A Pellizzon, Elisa Y Saito, João V Salvajoli

**Affiliations:** 1 Radiation Oncology Department, Hospital do Cancer A.C. Camargo, Sao Paulo, Brazil; 2 Pediatric Oncology Department, Hospital do Cancer A.C. Camargo, Sao Paulo, Brazil

## Abstract

**Purpose:**

To report our experience treating soft tissue sarcoma (STS) with high dose rate brachytherapy alone (HBRT) or in combination with external beam radiotherapy (EBRT) in pediatric patients.

**Methods and materials:**

Eighteen patients, median age 11 years (range 2 – 16 years) with grade 2–3 STS were treated with HBRT using Ir-192 in a interstitial (n = 14) or intracavitary implant (n = 4). Eight patients were treated with HBRT alone; the remaining 10 were treated with a combination of HBRT and EBRT.

**Results:**

After a median follow-up of 79.5 months (range 12 – 159), 14 patients were alive and without evidence of disease (5-year overall survival rate 84.5%). There were no local or regional failures in the group treated with HBRT alone. One patient developed distant metastases at 14 months and expired after 17 months. In the combined HBRT and EBRT group, there was 1 local failure (22 months), and 3 patients developed pulmonary metastatic disease 18, 38 and 48 months after diagnosis and no these patients were alive at the time of this report. The overall local control to HBRT alone and HBRT plus EBRT were 100 and 90%, respectively. The acute affects most common were local erythema and wound dehiscence in 6 (33%) and 4 (22%) patients.

Late effects were observed in 3 patients (16.5%).

**Conclusion:**

Excellent local control with tolerable side effects have been observed in a small group of paediatric patients with STS treated by HBRT alone or in combination with EBRT.

## Introduction

A variety of radiotherapeutic approaches have been used in the adjuvant local management of soft tissue sarcoma (STS). These include external beam irradiation (EBRT), brachytherapy (BRT), and intraoperative radiation therapy. Unfortunately, EBRT can cause growth retardation or adversely affect organ function in the pediatric population. Although randomized trials comparing BRT and EBRT in STS have not been published, hypothetically, brachytherapy offers several advantages for pediatric patients with soft tissue sarcoma (STS) over EBRT. BRT can reduce the dose of radiation to normal tissues and shortens the overall treatment time while maintaining a comparable high rate of local control. Thus, reductions in normal tissue doses decrease the probability of growth deformity, radiochemotherapy interactions, and theoretically, the rate of second tumor formation. BRT may also allow a reduction in the EBRT dose required [1]. So, BRT seems to be ideally suited for the pediatric patient when used alone or in combination with EBRT to achieve local control. Until early 90 decade, the reports enrolled patients treated with low dose rate isotopes. The local control rates were effective, but the operational difficulties to care children with brachytherapy sources make this approach restricted to few institutions. The value of HBRT for STS has been consistently demonstrated in adults with local control advantage of BRT over wide local excision alone for adults with high-grade tumors [1-5]. In children, limited data are available from series that include relatively small numbers of patients with different tumor types [6-10], however the theoretical advantages of HBRT makes this treatment modality an interesting option to multidisciplinary management of STS. In the current study we report our experience treating STS with HBRT in 18 patients who were submitted to BRT alone or in combination with EBRT. The clinical details of these patients and outcome are presented and discussed.

## Methods and materials

Eighteen pediatric patients with STS, median age 11 years (range 1 – 16 years), were treated with BRT between 1992 and 2004 at Hospital do Cancer A. C. Camargo. BRT was performed in conjunction with surgery, chemotherapy and EBRT during the initial management. Tables 1, 2, 3 contain pertinent clinical and treatment information obtained from the medical record. To ensure accuracy in reporting, disease status was confirmed for all patients prior to submission of the manuscript. No one was lost to follow up. The tumor size was determined from the gross pathologic description when available. It was otherwise obtained from the initial clinical description. Tumor grade, histology subtype, and margins were obtained from the microscopic pathologic description.

**Table 1:** Patient and treatment summary

Patient/age	Diagnosis	Grade*	Implant site	Margins	CMT	Group*	HDRBT (Gy)	EBRT (Gy)	Local failure	Distant failure	DFS (mo)
female/9	RMSE	III	Head neck	Negative	Yes	I	18	41.4			159
female/16	Synovial sarcoma	III	extremity	Negative	None	I	24	None			19
Female/2	ASPS	II	Extremity	Negative	Yes	I	24	None			21
Female/14	Synovial sarcoma	II	Extremity Hand	Positive	None	II	24	41.4		Lung	45
Male/5	Synovial sarcoma	II	Head neck	Negative	Yes	I	24	None			79
Female/12	ASPS	III	Extremity	Negative	None	I	18	30.6			29
Female/2	RMSE	II	Pelvic	negative	Yes	I	24	None			54
Male/4	ASPS	II	Extremity	Positive	Yes	II	24	None			143
female/1	ASPS	III	Extremity	Positive	Yes	II	30	None		Lung	18
Male/2	fibrosarcoma	II	Pelvic	Negative	Yes	I	40	None			83
Female/11	Synovia sarcomal	III	Extremity	Positive	Yes	II	24	50			141
Female/9	RMSE	III	Head Neck	Positive	Yes	III	18	43.2			65
Female/12	RMSE	III	Head Neck	positive	Yes	III	18	45			94
Male/2	ASPS	II	Pelvic	Negative	Yes	I	21	None			12
Male/9	Synovial sarcoma	II	Extremity	Negative	Yes	I	30	None			114
Female/12	RMS pleomorfic	II	Extremity	Negative	None	I	21	None			80
Female/3	Sarcoma indiferency	III	Extremity	Negative	Yes	I	21	41.4		Lung	38
female/16	ASPS	III	Extremity	Positive	Yes	III	24	50.0	yes	NCS	18

*Intergroup Rhabdomyosarcoma Study (IRS) staging used for both rhabdomyosarcomas and nonrhabdomyosarcomas. ASPS= soft tissue sarcoma alveolar, RMSE= Rhabdomyosarcoma embryonary

**Table 2:** Local control, distant failure, and survival rates according to margin, chemotherapy and treatment modality.

	**Local control (%)**	**Distant failure (%)**	**Overall survival (%)**
**Overall**	94.5 (17/18)	22 (4/18)	78 (14/18)
**Margins**			
Negative	100 (11/11)	1 (1/11)	91 (10/11)
Positive	85.7 (6/7)	42.8(3/7)	57.1 (3/7)
**Neoadjuvant chemotherapy**			
Yes	92.8 (13/14)	21.4(3/14)	78.5(11/14)
No	100(4/4)	25(1/4)	75 (3/4)
**Treatment modality**			
BRT alone	100 (8/8)	25 (2/8)	25 (2/8)
BRT+EBRT	90 (1/10)	20 (2/10)	20 (2/10)

**Table 3:** Institutional results for brachytherapy in pediatric tumors

**Center**	**Patients (n)**	**Brachytherapy**	**LCR (%)**
**St. Jude Children's Research Hospital (8)**	46	LDR	86
**University of Southern California (17)**	8	LDR	63
**Joint Center for Radiation Therapy (18)**	7	LDR (125Ir)	100
**Austrian data (16)**	12	Fract-HDR	75
**Ohio State University (14)**	12	Fract-HDR	91
**Ohio State University (21)**	13	IO-HDR	95
**Memorial Sloan-Kettering Hospital (15)**	10	IO-HDR	80
**Present study A.C. Camargo, Brazil**	18	Fract-HDR	94.5

### The brachytherapy procedure

BRT is the interstitial, intracavitary, or surface application of radioisotopes in a temporary or permanent fashion. All of the patients included in this study were treated with temporary interstitial implants using iridium-192 HDR microsource remote controlled by computer. Temporary implants were performed by placing afterloading catheters into the tumor bed most commonly, and some cases, after surgery, guide by an image procedure at the time of resection. Two techiniques were commonly used: intracavitary applicators and interstitial implants. Intracavitary brachytherapy was done by the confection of special devices (moulds) of catheter located into the vagina, nasopharynx and urethra, to treat of these sites. The moulds and catheter positioning were checked with orthogonal x-ray and the source position activated according to the isodose optimization. In the interstitial brachytherapy the tumor bed was jointly outlined by the surgeon and radiation oncologist. Permanent radiopaque clips were placed at the margins of the tumor bed. After loading, catheters were sutured into the tumor bed using chromic suture. One or both ends of the afterloading catheters were made to exit the site percutaneously at a short distance from the tumor bed. No effort was made to cover the wound or drain sites. In general, the catheters extended within the treatment plane 2 cm beyond the extent of the tumor bed. On the first postoperative day orthogonal plain-films were taken and the dosimetry of the treatment determined. The isotope and dose rates were selected to deliver 600 – 1000 cGy per day in two fraction eight hours between applications, with a minimum distance of 0.5 cm beyond the plane(s) of the implant. Dose distributions were calculated in multiple planes at 0.5- or 1.0-cm intervals that were roughly perpendicular to the ribbons. The highest dose rate for which the isodose contour was continuous and covers the CTV was selected as the prescription dose rate.

The dose rates were selected according to age, anatomic site and EBRT total dose received. The duration of the implant depended on the use of BRT as the only radiation modality or as a boost supplement to EBRT. BRT alone was generally used when tumor resection was complete with negative margins. The combination of EBRT and BRT was used for patients with involved margins. The mean number of catheters used per site was 6 (range 2–11) to cover a mean target volume of 59.7 cm3 (range 21 – 126).

### Statistical Analysis

Local failure was defined as tumor progression within the BRT volume. Regional failure was defined as tumor progression adjacent to and outside of the BRT or EBRT volume in the same organ or structure. Distant failure was defined as tumor progression in a previously noninvolved organ or structure. Overall survival was measured from the date of diagnosis. Disease-free survival was measured from the completion of radiation therapy, confirmed by biopsy or image exam. Kaplan-Meier method was used for survival analysis.

## Results

**Figure 1:**
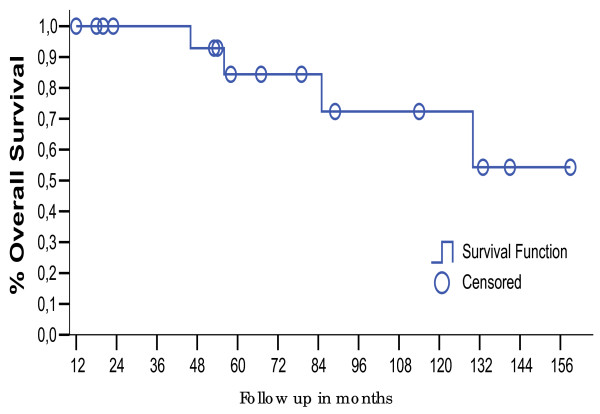
Overall survival for eighteen patients with STS treated with or without BRT.

**Figure 2:**
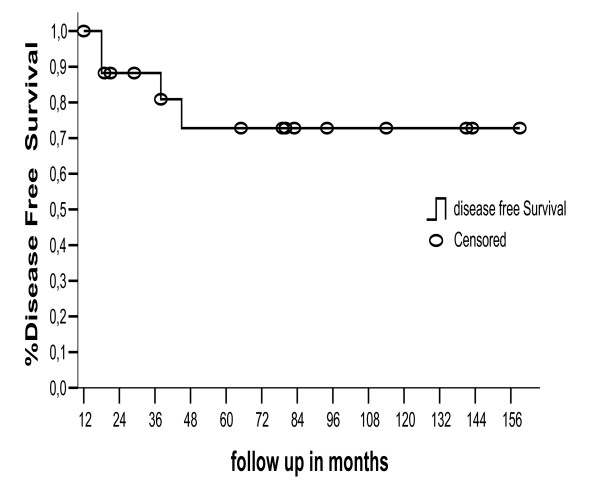
Disease Free Survival for patients with STS treated with EBRT+-BRT.

After a median follow-up of 79.5 months (range 12 – 159), 14 patients were alive and without evidence of disease. Overall survival rates at 5-year and 10-year was 84.4% and 72.4%, figure 1. 18 patients were initially managed with HBRT; HBRT alone was performed in 10 of these patients and the remainders were treated with a combination of HBRT and EBRT. The most common histologic subtypes include alveolar soft part sarcoma (n = 6), synovial cell sarcoma (n = 5), rhabdomyosarcoma embryonary (n = 5), fibrosarcoma (n = 1), indifference sarcoma (n = 1). All patients had intermediate to high grade tumors and most (n = 11) had no involved margins of resection at the time that HBRT was performed. intracavitary brachytherapy was done in 4 patients follow as: one vaginal, one urethral and two nasopharynx sites. There were no local or regional failures in the group treated with HBRT alone. One patient developed distant metastases at 45 months and expired after 85 months. Fourteen patients were alive with no evidence of disease 12, 19, 21, 29, 43, 56, 65, 79, 80, 83, 85, 94, 143 and 159 months after diagnosis. In the combined HBRT and EBRT group, there was 1 local failure (22 months), and 3 patients developed pulmonary metastatic disease 18, 38 and 45 months after diagnosis. The patients who presented with or who developed lung metastases were treated with pulmonary metastasectomy; no patients were alive at the time of this report. All patients died, including one of the 4 patients in this group who presented with nervous central system metastatic disease (table 1). Disease free survival at 5 was 72.4%, figure 2. The overall local control was 94.5% at time this report, in the group that was submitted to HBRT alone and HBRT plus EBRT local control was 100 and 90%, respectively. Patients who had gross total tumor resection without compromising of the margins had better local control compared to patients with positive margins (100% vs 85%), showed in table 2. The acute affects most common were local erythema present in 6 (33%) patients and wound dehiscence that occurred in 4 (22%) patients. Late effects were observed in 3 patients (16.5%). One 2-year old child with a vaginal rhabdomyosarcoma embrionary who received 24 Gy of HBRT and perioperative chemotherapy developed vaginal introitum stenosis, and corrected by genitoplasty procedure two years later. Another patient male of 11 years old with a synovial sarcoma in poplitea fossa who received.

HBRT with dose of 24 Gy and EBRT 50 Gy and perioperative chemotherapy, three years later developed muscle atrophy in the volume treated. A third patient male with 5 years old had a head neck synovial sarcoma and was treated by HBRT with dose of 24 Gy, one year later he developed dyschromy and teleangiectasy in area treated.

## Discussion

BRT may be used to deliver high doses of radiation in a localized volume, thereby reducing the probability of radiation-related side effects that are likely to occur when children are treated with external beam irradiation. The dose required to control STS exceeds that prescribed for the more common pediatric solid tumors which makes it more imperative that measures be taken to minimize the toxicity of radiation therapy and preserve function without compromising local control or overall survival.

In this context from 1982 we start to use BRT on management of STS, achieving satisfactory local control rate [11]. The introduction of HBRT on clinical practice makes this approach the preferential option to children brachytherapy after 1992 in our institution.

Hypothetically, fractionated HRBT combines the tissue-sparing, dosimetric advantages of brachytherapy treatment and the radiobiologic advantages of fractionation. Fractionated HRBT allows for reoxygenation and redistribution of residual cancer cells by delivering 300 cGy fractions twice daily to a total dose of 36 Gy during 8 days [12,13]. Repopulation is limited, because the RT is started within days of surgery and delivered through catheters implanted during surgery. At our institution, fractionated HBRT alone is reserved for those patients with limited volume disease, tumors with margins of resection negative. Patients with positive margins after ressection, high grade tumors, HBRT associated EBRT is used. Potter et al. [14] described their experience with fractionated HRBT in 12 children with primary and recurrent soft-tissue sarcomas, 7 of whom received HRBT as their sole treatment. The rate of local control and 2-year overall survival was 75% and 65%, respectively, with no significant morbidity [14]. Nag et al. [13] reported a 6-year actuarial local control rate of 91% and an overall survival rate of 81% in 12 children. Subcutaneous fibrosis and delayed dentition were noted in 2 of the children. Low-dose-rate brachytherapy (LBRT) has many advantages and is commonly used in adults for the treatment of soft-tissue sarcomas and other cancers. However, LBRT is difficult to administer in the pediatric population because of compliance issues and the potential radiation exposure to the caregivers. St. Jude Children's Hospital reported the use of LRBT alone or in combination with EBRT (range 12–60 Gy) in 46 patients with non-central nervous system malignancies [8]. Ours data are according to other series of the literature, table 3 lists the largest series to date dealing with brachytherapy in the pediatric population [8,13-17]. Parameters that are important to consider in choosing the appropriate intraoperative or perioperative technique include the presence of gross versus microscopic disease, the size and accessibility of the treatment volume, the position of adjacent critical structures, whether EBRT was previously given, and whether postoperative EBRT is planned. We use BRT combined with external beam irradiation for high-grade and intermediate-grade tumors with involved, inadequate, or indeterminate margins regardless of size or anatomic location. Low-grade tumors are treated with BRT only when the risk of recurrence and re-resection morbidity is high or at the time or recurrence.

High-grade adult tumors of all sizes treated with BRT alone have improved local control over those who receive no BRT when the tumor is grossly excised with or without involved margins [5]. There is a suggestion that adult patients with involved margins have a high probability of local control when treated with combined BRT and external beam irradiation compared to implant alone [1]. Tumor size and anatomic location have been debated as important factors to be used to guide decisions regarding the use of radiation therapy. Identifying prognostic factors and the relative indications for radiation therapy of STS in the pediatric population has been difficult to determine because of the histologic diversity and biology of these tumors and to identify subsets of patients who would not require irradiation and who would not be subjected to the long-term morbidity of irradiation. We have received children with tumors generally smaller than those found in adults, that wide local excision likely produced substantial morbidity, in this way wide local excision is often not possible, because these patients often lack subcutaneous tissue. Further, patients are commonly referred to our institution following limited, non oncologic resection in the community which further confounds our ability to differentiate or identify patients who may be treated with surgery alone. In these cases BRT alone or in combination EBRT may be used to deliver high doses of radiation in a localized volume, thereby reducing the probability of radiation-related side effects. It is difficult to retrospectively evaluate the impact of HBRT on structure and function. The rate of complication, both acute and chronic, ranges from 10–48% depending on the series [18,19]. In our patients, 4 suffered wound dehiscence after HBRT; one received preimplant EBRT, the other three were treated with chemotherapy in the perioperative period. The most common side effects of HBRT were radiation local erythema, teleangiectasy and fibrosis that are likely to occur when children are treated with external beam irradiation alone.

In conclusion, excellent local control with tolerable side effects have been observed in a small group of paediatric patients with STS treated by HBRT alone or in combination with EBRT. Younger patients with STS may achieve local control and prevent growth retardation with a combination of BRT and moderate doses of EBRT. Longer follow-up is required to determine the full extent of late effects. Limb preservation, functional outcome, and toxicity assessment require careful assessment in a prospective study.

## Competing interests

The authors declare that they have no competing interests.

## Authors' contributions

GAV carried out the search, acquisition and interpretation of the data in studies. GAV performed the statistical analysis and drafted the manuscript. PEN participated in the design of the study, carried out the search for articles and gave final approval of the version to be published. AAJ and EYS participated in the design of the study, JVS gave final approval of the version to be published, ACAP gave final approval of the version to be published, CBA participated in the design of the study and gave final approval of the version to be published. All authors read and approved the final manuscript.

## References

[B1] Alekhteyar KM, Leung DM, Brennan MF (1996). The effect of combined external beam radiotherapy and brachytherapy on local control and wound complications in patients with high grade soft tissue sarcomas of the extremity with positive microscopic margin. Int J Radiat Oncol Biol Phys.

[B2] Brennan MF, Hilaris BS, Shiu MH (1987). Local recurrence in adult soft tissue sarcoma – a randomized trial of brachytherapy. Arch Surg.

[B3] Demetri GD, Pollock R (1998). NCCN Sarcoma practice guidelines. Oncology.

[B4] Habrand JL, Gerabaulet A, Pejovic MH (1991). Twenty years experience of interstitial iridium brachytherapy in the management of soft tissue sarcomas. Int J Radiat Oncol Biol Phys.

[B5] Harrison LB, Franzese F, Gaynor JF (1993). Long term results of a prospective randomized trial of adjuvant brachytherapy in the management of completely resected soft tissue sarcomas of the extremity and superficial trunk. Int J Radiat Oncol Biol Phys.

[B6] Cherlow JM, Syed AM, Puthawala A (1990). Endocurietherapy in pediatric oncology. Am J Pediatr Hematol Oncol.

[B7] Curran WJ, Littman P, Raney RB (1988). Interstitial radiation therapy in the treatment of childhood soft-tissue sarcomas. Int J Radiat Oncol Biol Phys.

[B8] Fontanesi J, Rao BN, Fleming ID (1994). The St. Jude Children's Research Hospital experience. Cancer.

[B9] Gerabaulet A, Panis X, Flamant F (1985). Iridium afterloading curietherapy in the treatment of pediatric malignancies: The Institut Gustave Roussy experience. Cancer.

[B10] Haie-Meder C, Flamant F, Revillon Y (1994). The role of brachytherapy in the therapeutic strategy of vesico-prostatic rhabdomyosarcoma in children. Ann Urol (Paris).

[B11] Novaes PERS (1985). Interstitial therapy in the management of soft-tissue sarcomas in childhood. Medical and Pediatric Oncology.

[B12] Nag S, Tippin D, Ruymaan FB (2001). Intraoperative high-dose-rate brachytherapy for the treatment of pediatric tumors: The Ohio state university experience. Int J Radiat Oncol Biol Phys.

[B13] Nag S, Martinez-Monge R, Ruymann FB (1997). Innovation in the management of soft tissue sarcomas in infants and young children: High-dose-rate brachytherapy. J Clin Oncol.

[B14] Potter R, Knocke TH, Kovacs G (1995). Brachytherapy in the combined modality treatment of pediatric malignancies: Principles and preliminary experience with treatment of soft tissue sarcoma (recurrence) and Ewing's sarcoma. Klin Padiatr.

[B15] Merchant TE, Zelefsky MJ, Sheldon JM (1998). High-dose-rate intraoperative radiation therapy for pediatric solid tumors. Med Pediatr Oncol.

[B16] Cherlow JM, Nisar Syed AM, Puthawala A (1990). Endocurietherapy in pediatric oncology. Am J Pediatr Hematol Oncol.

[B17] Healey EA, Shamberger RC, Grier HE (1995). A 10-year experience of pediatric brachytherapy. Int J Radiat Oncol Biol Phys.

[B18] Arbeit J, Hilaris BS, Brennan MF (1987). Wound complications in the multimodality treatment of extremity and superficial truncal sarcomas. J Clin Oncol.

[B19] Ormsby M, Hilaris BS, Nori D (1989). Wound complications of adjuvant radiation therapy in patients with soft tissue sarcomas. Ann Surg.

